# Supplementary figures and images for: Divergent Plasmodium actin residues are essential for filament localization, mosquito salivary gland invasion and malaria transmission
Source: PLoS Pathog. 2022 Aug 23;18(8):e1010779. doi: 10.1371/journal.ppat.1010779 (PMC9439217; doi:10.1371/journal.ppat.1010779)

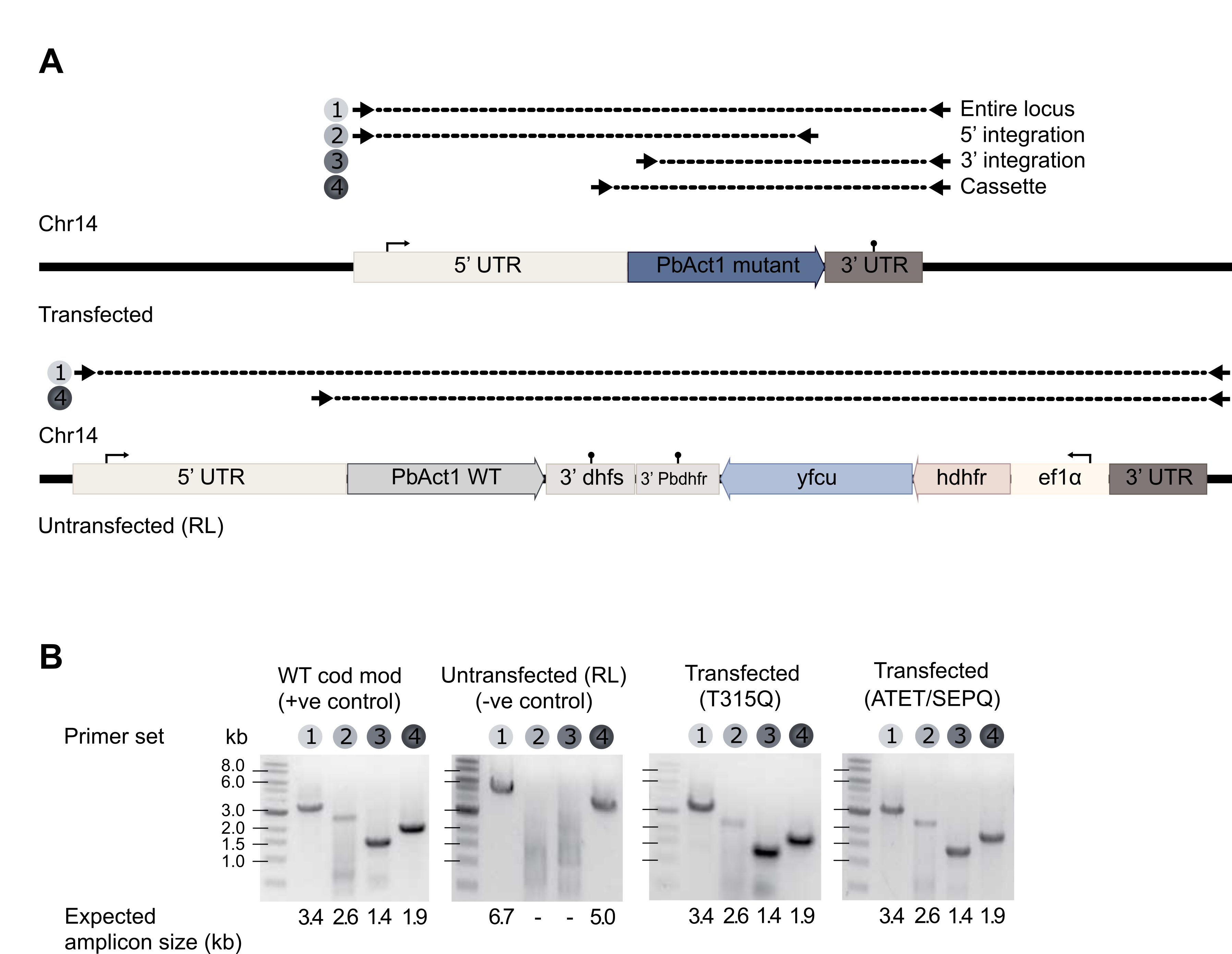

Supplement: S1 Fig — (A) Integration schemes and the arrangement of the genomic locus in both untransfected (recipient line, RL, below) and transfected lines. The numbers indicate the primer combination used (see S1 Table) and the relative positions of those primers. (B) Representative gels of selected genotyped parasite lines. (TIF) [file ppat.1010779.s001.tif]

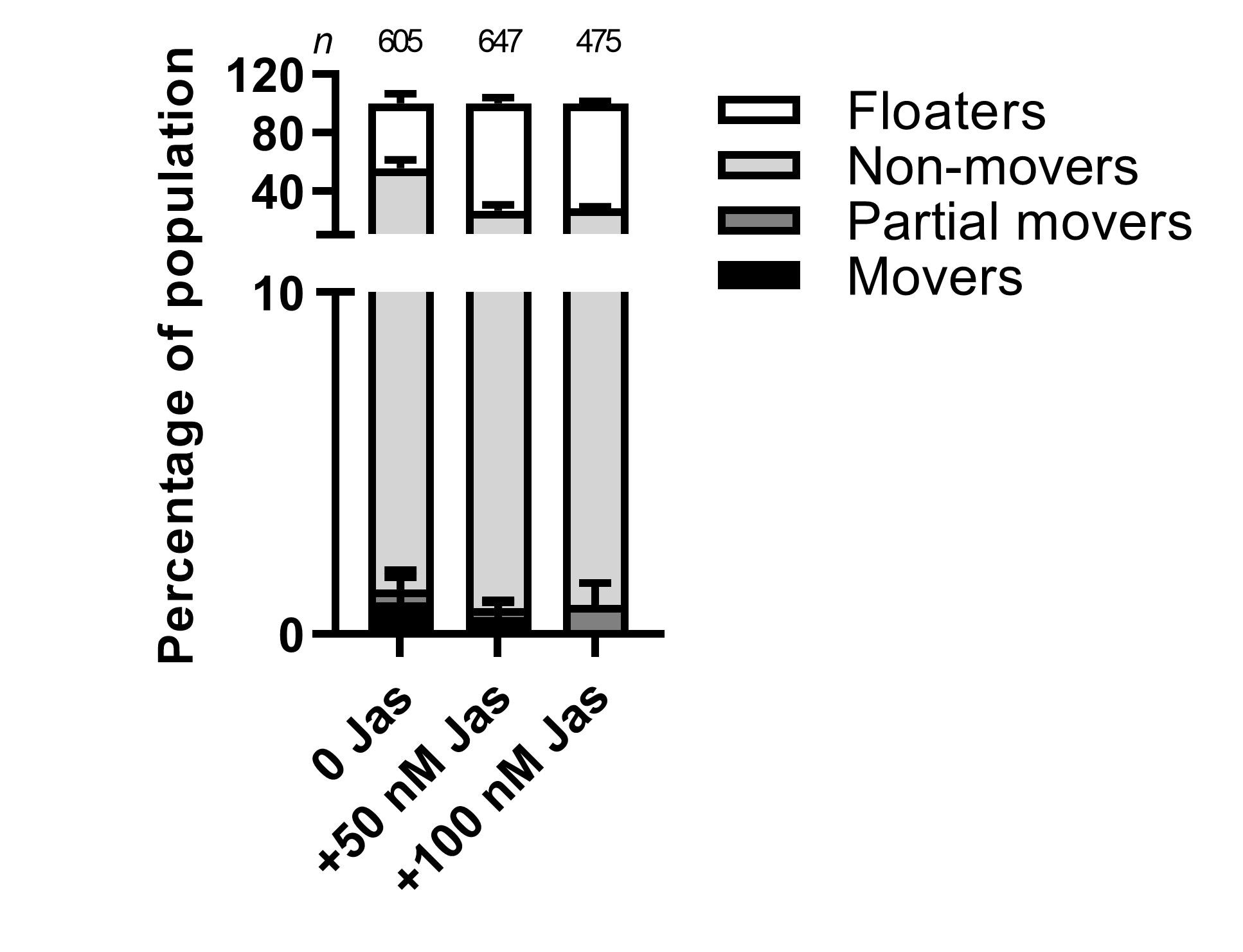

Supplement: S2 Fig — Hemolymph sporozoites were extracted and activated as described in the Material and Methods section. Low concentrations of Jas were added after several movies (without Jas) were acquired. Addition of Jas did not improve motility. (TIF) [file ppat.1010779.s002.tif]

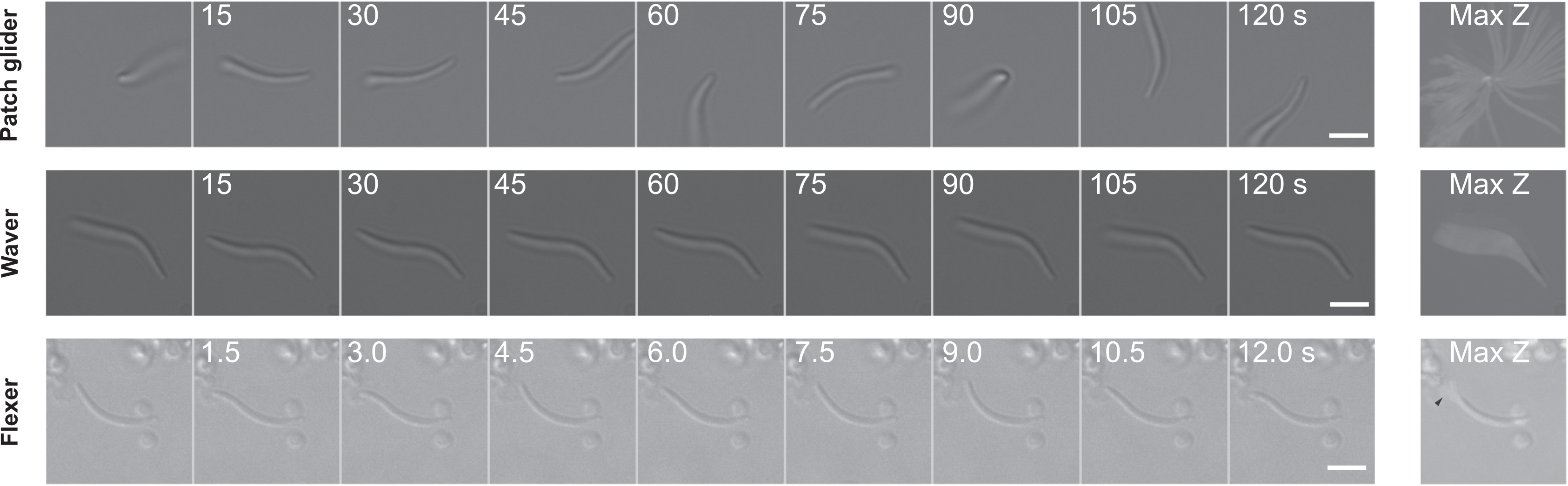

Supplement: S3 Fig — (TIF) [file ppat.1010779.s003.tif]

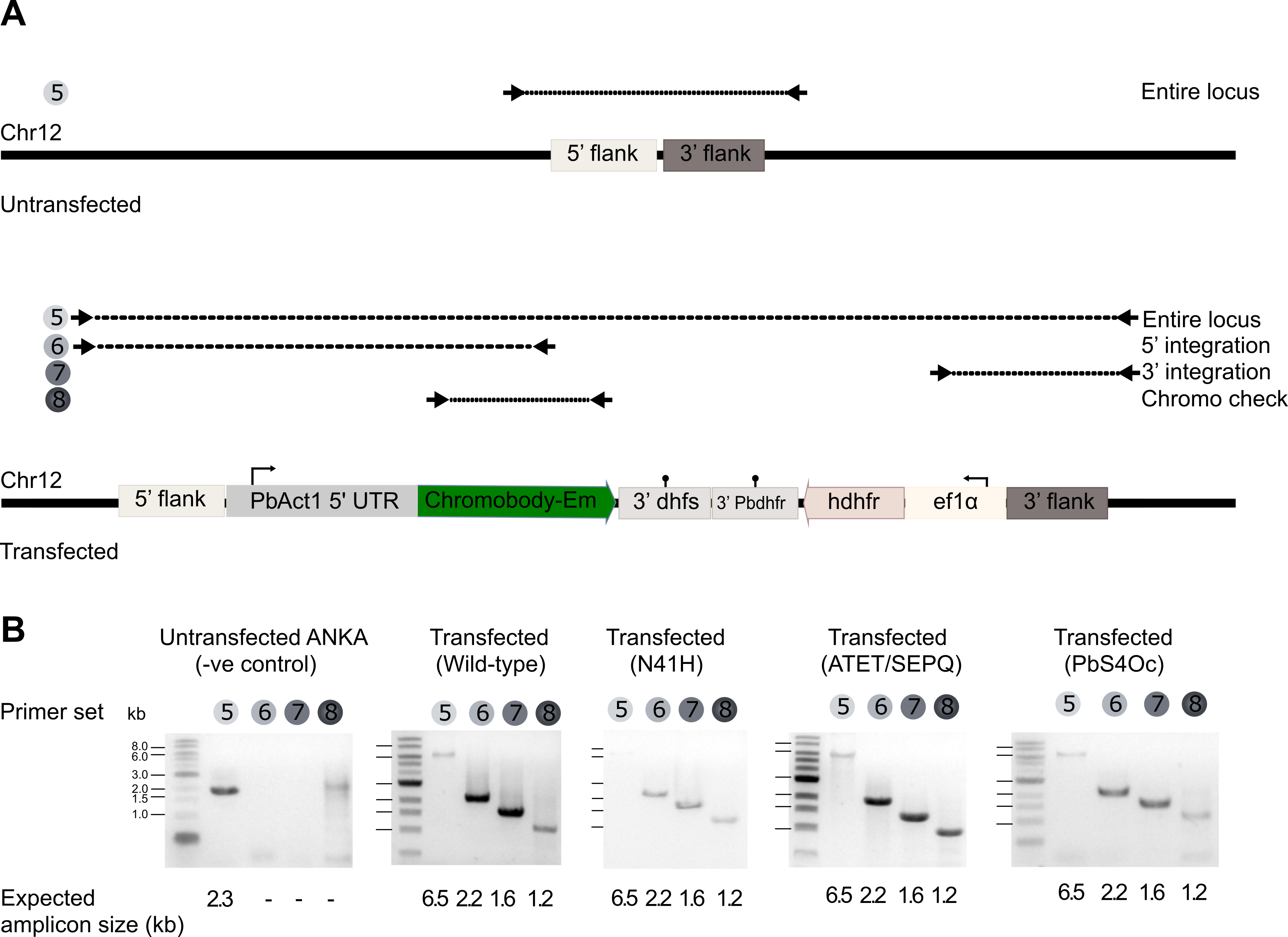

Supplement: S4 Fig — (A) Integration schemes and the arrangement of the genomic locus in both untransfected and transfected lines. The numbers indicate the primer combination used (see S1 Table) and the relative positions of those primers. (B) Agarose gel electrophoresis of generated isogenic lines. (TIF) [file ppat.1010779.s004.tif]

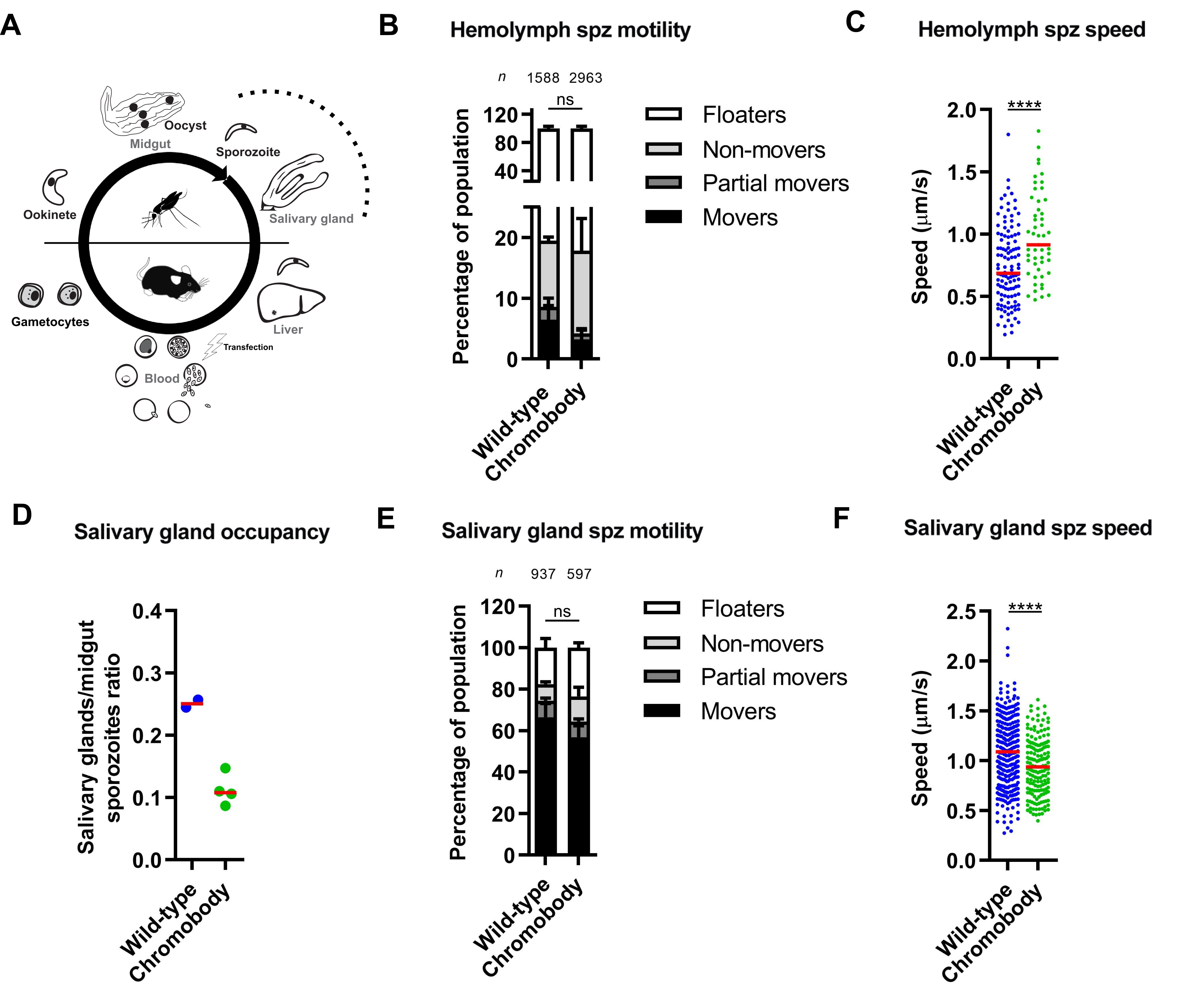

Supplement: S5 Fig — (A) Schematic representation of the Plasmodium life cycle with late mosquito stages indicated with a dashed line. (B) Hemolymph sporozoite motility patterns were analysed using a 2D motility assay and revealed no significant differences to a parallel control background cage. Bars represented as mean ± standard deviation for repeats between at least two technical repeats from a single infection each. Fischer’s exact test. (C) Speeds of moving hemolymph sporozoites indicate that chromobody sporozoites might move slightly faster, yet were in comparable ranges and likely represent experimental variations despite being significantly different. Red line indicates median value. Mann-Whitney test, ****p<0.0001. (D) The actin chromobody line displays a moderate decrease in salivary gland invasion. Red line indicates median value. Dots represent technical repeat counts from day 18 and day 19 post-infection (one mosquito infection for the control line and two independent infections for the actin chromobody line). (E) Salivary gland sporozoites are similarly motile between control and chromobody lines. Bars represented as mean ± standard deviation for repeats between at least two technical repeats. Fischer’s exact test. (F) Actin chromobody expressing salivary gland sporozoites showed similar range of speeds when compared to the wild-type control but were significantly slower on average. Values are in the normal range for wild-type parasites and likely represent experimental variations despite being significantly different. Red line indicates median value. Mann-Whitney test, ****p<0.0001. (TIF) [file ppat.1010779.s005.tif]

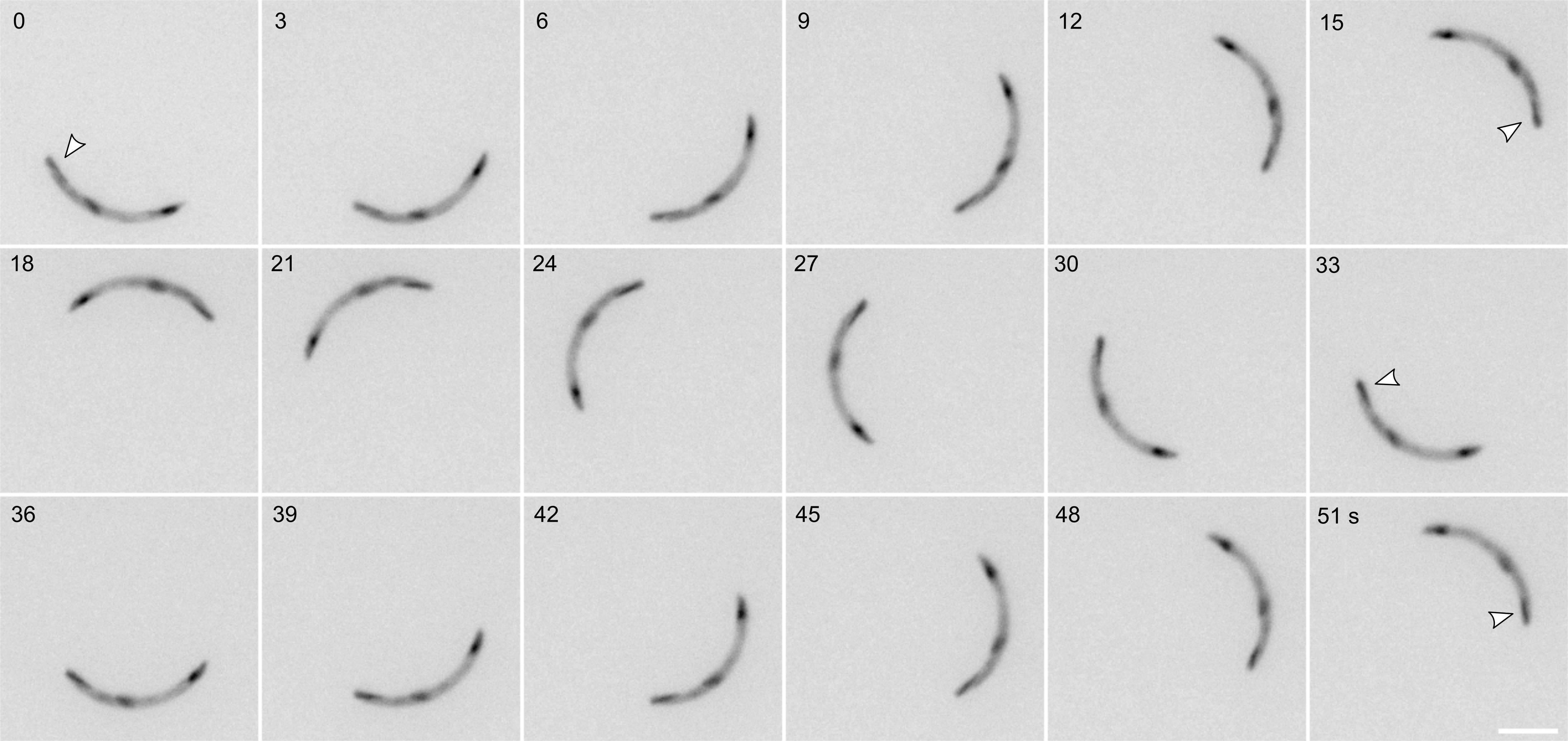

Supplement: S6 Fig — The white arrowhead indicates the rear of the sporozoite. In this case, a noticeable signal develops at the back with time. Scale bar: 5 μm. Changes in signal distribution were observed infrequently. (TIF) [file ppat.1010779.s006.tif]

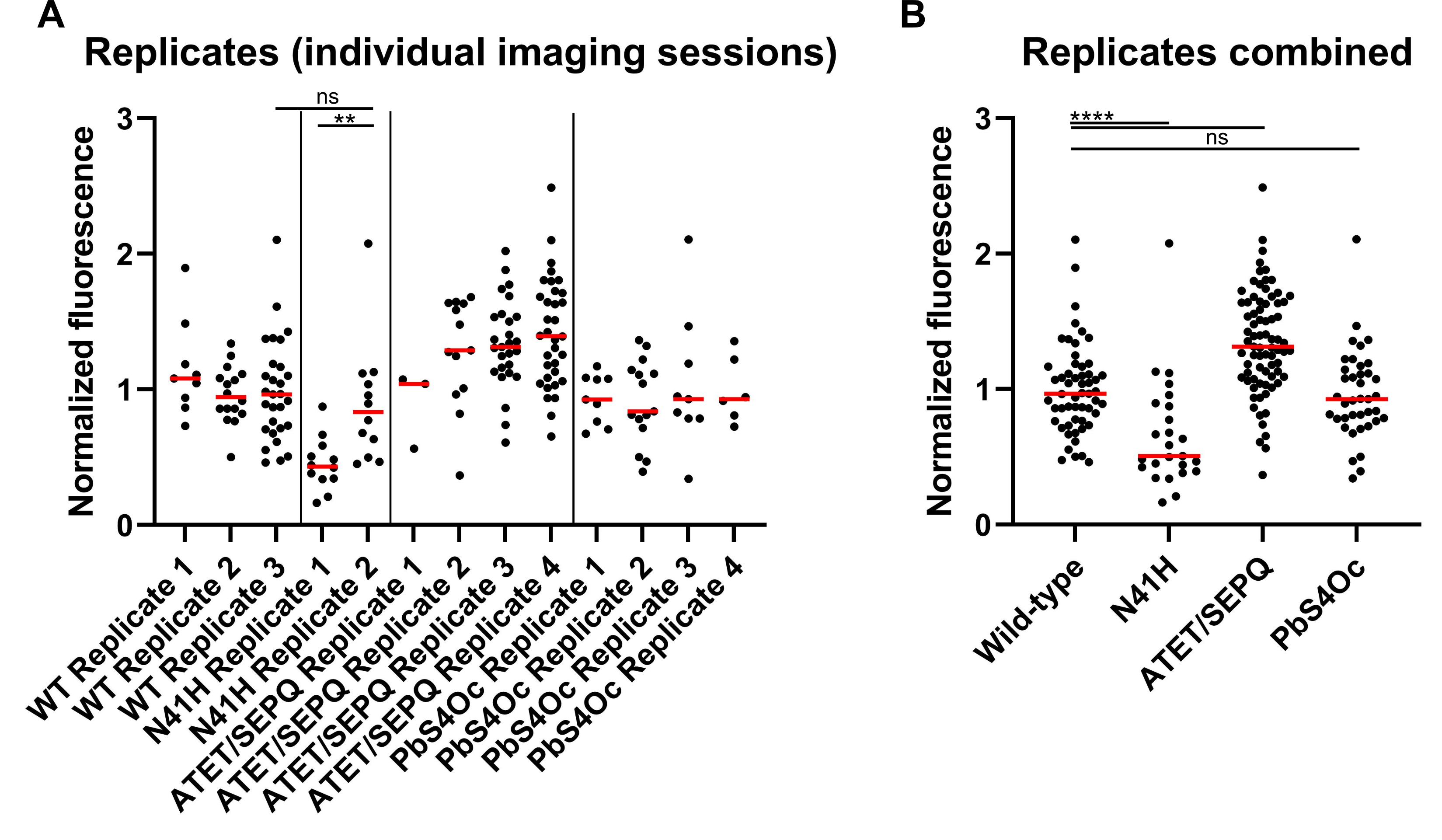

Supplement: S7 Fig — (A) Individual readings from separate replicates (individual imaging sessions) for each parasite line. Each data point reflects a background subtracted front and back sporozoite intensity normalized to the average wild-type control. Note that N41H median intensities were variable between duplicate imaging sessions. (B) Data of replicates in (A) combined. Red line indicates median. Mutants generally did not have major changes in their normalized fluorescence levels, with the exception of N41H which might have reduced functional protein levels. Mann-Whitney test, **p = 0.001 ****p<0.0001. ATET/SEPQ had a minor increase in intensity that likely reflects assay limitations and experimental variation despite being statistically significant. (TIF) [file ppat.1010779.s007.tif]
