# Supplementary material for: Divergent Plasmodium actin residues are essential for filament localization, mosquito salivary gland invasion and malaria transmission
Source: PLoS Pathog. 2022 Aug 23;18(8):e1010779. doi: 10.1371/journal.ppat.1010779 (PMC9439217; doi:10.1371/journal.ppat.1010779)
Supplement: S1 Table — (DOCX) [file ppat.1010779.s009.docx]

| ***Plasmid generation – actin 1 mutagenesis*** | |  | | |  |  | |
| --- | --- | --- | --- | --- | --- | --- | --- |
| **Primer no.** | **Primer name** | **Sequence (5'-3')** | | |  |  | |
| 1 | N41H F | GGACGTCCGAAACATCCGGGTATTATGG | | |  |  |  |
| 2 | N41H R | CCATAATACCCGGATGTTTCGGACGTCC | | |  |  |  |
| 3 | A272S F | CTGGGTATGGAATCAGCAGGTATTCATACC | | |  |  |  |
| 4 | A272S R | GAATTCCTGCTGATTCCATTCCTAAAAAGG | | |  |  |  |
| 5 | T277E F | GCAGGTATTCATGAAACCACCTTTAAC | | |  |  |  |
| 6 | T277E R | GTTAAAGGTGGTTTCATGAATACCTGC | | |  |  |  |
| 7 | E308P F | GCACCACCATGTATCCAGGTATTGGTGAACG | | |  |  |  |
| 8 | E308P R | CGTTCACCAATACCTGGATACATGGTGGTGC | | |  |  |  |
| 9 | T315Q F | GGTGAACGCCTGCAACGTGATATTACC | | |  |  |  |
| 10 | T315Q R | GGTAATATCACGTTGCAGGCGTTCACC | | |  |  |  |
|  |  |  | | |  |  | |
| ***Genotyping primers- actin 1 replacement (see Fig S1)*** | | | | |  |  | |
| **Primer no.** | **Primer name** | **Sequence** | | | **Used for** |  | |
| Combination 1 | 5' UTR flanking F | GTGCTCATAAGATAATAACTTCA | | | Entire integration genotyping |  | |
|  | 3' UTR flanking R | GTGATTGGGTTTTTCGTACTAG | | |  |  | |
| Combination 2 | 5' UTR flanking F | GTGCTCATAAGATAATAACTTCA | | | 5' integration genotyping |  | |
|  | Actin codon modified R | CTGAAAGGTGCTCAGGCTGC | | |  |  | |
| Combination 3 | Actin codon modified F | CAATTCAGGCAGTTCTGAGCC | | | 3' integration genotyping |  | |
|  | 3' UTR flanking R | GTGATTGGGTTTTTCGTACTAG | | |  |  | |
| Combination 4 | Actin promoter 3’ end F | AAGGTACTTTTCATCGTGC | | | Cassette check |  | |
|  | 3' UTR flanking R | GTGATTGGGTTTTTCGTACTAG | | |  |  | |
|  |  |  | | |  |  | |
| ***Plasmid generation – actin chromobody*** | | | | |  |  | |
| **Primer no.** | **Primer name** | **Sequence (5'-3')** | | |  |  | |
| 11 | Chromobody NotI F | aaggaaaaaagcggccgctatggctcaggtgcagc | | |  | |  |
|  | | | |  |  |  |  |
| 12 | Emerald BamHI R | CGCGGATCCTTACTTGTACAGCTCGTCCATGC | | |  | |  |
|  | | | |  |  |  |  |
|  | | | |  |  |  |  |
|  |  |  | | |  |  | |
| ***Genotyping primers- actin chromobody-emerald (see Fig S4)*** | | | | |  |  | |
| **Primer no.** | **Primer name** | **Sequence** | | | **Used for** |  | |
| Combination 5 | Chr12 5’ flank F | GTAGGTCGACCCGTATCTTATATAATGACGTAGCAATATATCATTC | | | Entire integration genotyping |  | |
|  | Chr12 3’ flank R | GTAGCTCGAGGATGATTTAGAATCTTTATATGCACCTATGC | | |  |  | |
| Combination 6 | Chr12 5’ flank F | GTAGGTCGACCCGTATCTTATATAATGACGTAGCAATATATCATTC | | | 5' integration genotyping |  | |
|  | Actin 5' UTR R | GCTCTAGATTTAATTTTTTTTTTAAGTATATGAGTATATATATGTGTGTAAAAATTTATATTAAATATGC | | |  |  | |
| Combination 7 | ef1alpha promoter F | GAAATATAAATAATTACGCCTAGTTAATAAAGGGCAC | | | 3' integration genotyping |  | |
|  | Chr12 3’ flank R | GTAGCTCGAGGATGATTTAGAATCTTTATATGCACCTATGC | | |  |  | |
| Combination 8 | Chromobody F | AAGGAAAAAAGCGGCCGCTATGGCTCAGGTGCAGC | | | Chromobody-emerald check |  | |
|  | Emerald R | AGCCCTAGGTTACTTGTACAGCTCGTCC | | |  |  | |
|  | | |  |  |  |  |  |
|  | | |  |  |  |  |  |
|  | | |  |  |  |  |  |
|  | | |  |  |  |  |  |
