# Supplementary material for: Divergent Plasmodium actin residues are essential for filament localization, mosquito salivary gland invasion and malaria transmission
Source: PLoS Pathog. 2022 Aug 23;18(8):e1010779. doi: 10.1371/journal.ppat.1010779 (PMC9439217; doi:10.1371/journal.ppat.1010779)
Supplement: Attachment — Submitted filename: Yee et al_Responses to reviewer comments_with tech changes.pdf [file ppat.1010779.s010.pdf]

Appendix 1: Responses to editor and reviewer comments

Comments from editor

In addition to the reviewers' comments, it would be helpful for the readers to include a more detailed figure showing the contacts made by the residues that have been mutated in this study in the actin filament. In figure 1A, it would be better to have colors that are easier to distinguish from each other.

Authors' response: We have now added insets to show close-ups of relevant regions within the filament (see revised Figure 1A and B). As published in the high-resolution structure of *Plasmodium* actin 1, there are surprisingly few direct contacts between monomers within the filament (Pospich *et al.* 2017 Proc Nat Acad Sci 114(49):10636). However, changes in chemical nature of these amino acid residues could result in altered interaction profiles that could affect actin dynamics. We have thus also added text emphasizing that changes of these residues between species could have consequences for intra- and inter-molecular contacts and subsequent filament dynamics (lines 160-165 of clean version and Figure 1 legend).

Author's responses to reviewer comments

Part I - Summary

Reviewer #1: Yee et al explore the functional role of divergent Plasmodium actin residues. Previous work showed that subdomain 1 and 4 of Plasmodium actin contributed to motility in mosquito stage parasites. Subdomain 2 and 3 proved essential because these domains could not be exchanged with the mammalian sequence. Here the sequences of subdomains 2 and 3 were compared with mammalian sequences and specific conserved residues of interest were identified based on three well justified criteria. Two residues in domain two were mutated to the conserved mammalian residue, but only N41H could be recovered. Five residues in subdomain three were mutated, but only four were recovered. A272S, T277E, E308P, and T315Q. In addition to the single point mutants a quadruple subdomain 3 mutant ATET/SEPQ was generated. The authors then infected mosquitos with mutant gametocytes and assessed oocyst levels in the mosquito midgut. A272S had a modest reduction in oocyst levels, but the quadruple mutant was severely diminished indicating that subdomain 4 plays an important role in mosquito specific actin dynamics. This interpretation is supported by dramatically reduced ookinete motility. Next the authors examined the ability of sporozoites to establish infection in the salivary gland. Compared to the control all the point mutants had reduced salivary gland occupancy. Subdomain 2 N41H mutants isolated from hemolymph and salivary glands was found to have reduced motility. The subdomain 3 E308P mutant was also found to have reduced motility but the quadruple mutant could not be examined because only two sporozoites were observed. The authors tested the ability of the mutants to be transmitted. Natural transmission was found to be lower as expected due to reduced salivary gland parasite loads, but i.v. mouse infections proceeded similar to the controls indicating that the substitutions found in Plasmodium actin are likely present to optimize mosquito stage infection. To gain further insight into how the point mutants impact actin dynamics the authors utilized an actin chromobody to visualize actin. They found an asymmetric localization of actin on one side of mobile sporozoites that could be disrupted with either CytoD (even distribution of the chromobody) or Jas (localization to both ends). These changes in distribution allow for interpreting the impact of the substitutions in terms of stabilizing or destabilizing actin filaments. Introduction of the actin chromobody into N41H led to chromobody accumulation at both ends of the cell indicating that this mutation stabilizes actin. The ATET/SEPQ mutant resembled CytoD treated cells indicating that the mutations led to destabilized actin

perhaps due to loss of actin binding protein interactions. The manuscript is well organized and well written. The work convincingly demonstrates that finely tuned actin dynamics are essential for efficient organ entry into the mosquito and ultimately for transmission.

Reviewer #2: The overall goal of this study is to assess the effect of several individual (or a group of 4) point mutants in Plasmodium actin 1 at four different stages of the parasite lifecycle, encompassing both the mosquito and the rodent hosts. Actin is generally highly conserved, but Plasmodium actin 1 has regions that are quite divergent. Mutation of a subset of these divergent residues in subdomains 2 and 3 to the more commonly found amino acid at that positon resulted in viable parasites that were selected for further phenotypic investigation. Interestingly, some of the major effects of the mutations were seen in transmission to and through the insect, and in particular resulted in impairment in salivary gland invasion, suggesting that this step is potentially a difficult barrier that the parasite must overcome to complete its lifecycle. A novel feature of this study is the use of the actin-chromobody for visualization of actin in sporozoites (Plasmodium actin has been notoriously difficult to visualize until quite recently). I find the study thoughtfully done and interesting. It is both a logical follow-up of their earlier studies with actin subdomain swaps, and also provides a roadmap for future investigation of several of these mutations with regard to how they might affect interaction with actin-binding proteins and thus alter filament dynamics.

Reviewer #3: This work reports in vivo effects of actin mutations in Plasmodium at different life stages. Previous studies have shown that actin is essential for Plasmodium and only lethal mutants had been characterized. The authors focused on actin residues that are different from mammalian actins and found several point mutations that keep the parasite viable but cause defects in their behaviors. Their analysis suggests different requirements of actin filament dynamics at each life stage. They also utilized actin chromobody to visualize actin localization in motile sporozoite, which should become a very useful tool for future cytoskeletal research in Plasmodium. Overall, this research is well done with quantitative analysis and careful interpretation and should provide significant advances to the related field.

Authors' response to all the above reviewer's assessments: We would like to thank the reviewers for their accurate summaries of our manuscript and for the constructive and positive assessment of our submitted work.

Part II – Major Issues: Key Experiments Required for Acceptance

Reviewer #1: Figure 5. Many actin probes have an impact on actin dynamics which the authors show has a role in Plasmodium transmission. A control experiment measuring the speed of movement such as in Fig 3 C and F for true WT sporozoites versus ones expressing the actin chromobody would reveal whether actin dynamics are appreciably altered by the chromobody. The use of probes is necessary in order to visualize how different conditions impact actin localization, so the aim would be to determine the level to which the chromobody impacts actin dynamics.

Authors' response: We have now provided detailed characterization of the actin chromobody line in terms of sporozoite behaviour in comparison to a background control line fed at a similar time period (see new Supplementary Figure 5). This indicated that the expression of this probe had minimal effects on the pattern of sporozoite motility but had a minor yet measurable effect on parasite speed. Interestingly, we observed that there was

a moderate decrease in the amounts of salivary gland sporozoites for the chromobody line, indicating a possible effect of chromobody expression on salivary gland invasion. This further emphasizes just how sensitive salivary gland invasion is to minor alterations of its actin dynamics. This has been pointed out in the results text (lines 265-269) and included in our revised discussion (lines 361-365 and 405-409 in clean version of the manuscript).

Figure 6. The authors only examined the ATET/SEQP mutant for subdomain 3. They found that together these mutations resulted in actin destabilization. We are left to wonder if each residue is contributing to destabilization or if there might be some residues that are more important as suggested by the single mutant analysis in Figures 1-4. Therefore, the authors should complete their analysis by examining chromobody localization in each of the single mutants of subdomain 3. This would allow some correlation with the speed of movement reported in Fig 3C and F.

<u>Authors' response:</u> We agree that it would be interesting to delineate the contributions of each subdomain 3 amino acid residue to filament destabilisation using the actin chromobody and have discussed this among the authors during the study. We came, however, to the conclusion that this would be (i) too much work for the potential insights gained and hence (ii) hard to justify the large numbers of mice that would need to be sacrificed for such experiments. We therefore kindly ask for the reviewer's understanding that this goes well beyond the scope of the current study and also timescale for a revision. We would need to generate multiple chromobody lines to properly characterize the contribution: four single mutants, plus combinations of various double and triple mutants. We anticipate the need to produce double and triple mutants, since none of the single subdomain 3 mutants have a significant effect on hemolymph sporozoite motility (in terms of movers in the population- the primary basis of our selection for chromobody analysis), so we strongly suspect that this is a combined residue effect on actin dynamics which ultimately affects motility. Such an effect was also seen for another actin subdomain (Douglas et al 2018 PLOS Bio 16(7):e2005345). We anticipate that it would take more than a year to do this characterization properly with many more mice.

Reviewer #3: 1. Point mutations can often affect the protein levels due to changes in protein stability in vivo. If the protein level is significantly altered, it can have an impact on the phenotype. Therefore, the authors should confirm that the actin protein levels were not altered in the mutants that they analyzed. It is not clear whether an actin antibody is available to detect Plasmodium actin on Western blot. Even if it is not available, actin should be a major 42 kDa protein and could be estimated from an SDS-PAGE gel.

<u>Authors' response:</u> Antibodies used against *Plasmodium* actin 1 are unfortunately not reliable for any careful quantitation in sporozoites as they are not very specific and/or sensitive. This poses indeed a technical limitation to our system and warrants further work in our field.

We did not anticipate major protein level differences as, for the majority of the mutants, phenotypes were not observed at other parasite life cycle stages. Major protein level differences would clearly have consequences elsewhere and this was not observed. We are also confident that our mutants are not misfolded since, in our chromobody lines, addition of Jasplakinolide resulted in the collection of signal on both ends of the sporozoite indicating that the mutant actin readily incorporates similar to wild-type actin and is therefore functional for polymerization. Indeed, mutations in some positions of interest for this study published previously did not result in misfolding based on the structures determined (Kumpula et al 2019 PLOS Bio 17(6):e3000315).

However, to attempt to more directly estimate relative actin protein levels of sporozoites, we quantified the chromobody fluorescence signal of each available line after Jasplakinolide treatment. These lines represent every mutation of interest in this study. Background subtracted combined intensities of the front and back for multiple sporozoites were normalized and compared to the wild-type control. This could serve as a very crude proxy for functional actin levels (since they can incorporate into filaments) and we have added this analysis to the supplement (see new Supplementary Figure 7). By applying this analysis across multiple imaging sessions, we observed that mutations generally did not have a major effect on actin levels, with the exception of the mutant N41H, which might have decreased levels. We have indicated the results of this analysis and the potential decrease in N41H levels in the results (lines 299-303 of the clean version of this manuscript). This observation is nonetheless striking in terms of our understanding of this mutant since, even in the event of potentially decreased protein levels, N41H still showed a localization resembling Jasplakinolide treated wild-type sporozoites and hence more (or more stable) filaments. Given the critical effect of concentration on actin polymerization, the data together emphasize that this mutation renders a more "polymerization-prone" actin *in vivo*. We have added this observation to the discussion to highlight this point (lines 454-462 of the clean version of this manuscript).

Part III – Minor Issues: Editorial and Data Presentation Modifications

Reviewer #1: Line 1018 remove "infections"

Authors' response: The accidental addition of this word has been removed.

Mammalian actin is likely optimized for function at 37C. Mosquitos are grown at cooler temperatures. It is known that actin point mutations can alter sensitivity to temperature. It seems that Plasmodium actin has to be optimized for both conditions, but this was not discussed. It seems that all the motility studies were performed at room temperature. If the authors had access to a temperature controlled stage it would be interesting to test if motility of the mutants can be rescued with higher temperatures. This would help explain why infectivity defects are present in mosquitos but not in mice.

Authors' response: Temperature changes is a very interesting discussion point and one that we have spent much time thinking about. Indeed, the parasite is exposed to different temperatures in which its actin dynamics need to cope efficiently and we touched on this point in our previous publication (Douglas et al 2018 PLOS Bio 16(7):e2005345). We have shown previously that at higher temperature sporozoites move faster but for a shorter time (Hegge et al 2009 Biotech J 4:903). As speed reversely correlates with sporozoite adhesion we postulate that adhesion turnover is increased with increasing temperatures, but have not studied this yet. Indeed, this would be interesting to formally investigate with a selected number of mutants. In the case of the present study, however, temperature does not seem to be the major necessity for efficient motility since almost all single mutants glide comparable to wild-type when isolated from the hemolymph or salivary glands at speeds that are in a similar range to speeds of sporozoites in warm skin (Amino et al 2006 Nature Med 12(2):220, Bane et al 2016 PLOS Pathog 12(7):e1005710). Yet, these mutants struggle to invade the salivary glands. The issue therefore seems to be specifically in penetration of the salivary glands, which would naturally occur at a fairly typical room temperature and not 37℃. Assessing the effect on temperature on salivary gland invasion (to see if this could rescue to phenotype) is unfortunately not possible as *P. berghei* is poorly infectious when mosquitoes are housed at temperatures above 21℃.

In line with the reviewer's comment, we nonetheless think it is important to include discussion on the point of temperature variation between hosts and have included this in

lines 391-395 of the clean revised manuscript version. This highlights an important consideration that the parasite has to overcome in order to effectively transmit.

Jas stabilizes actin filaments, it would be interesting to test if low doses of Jas could rescue the speed of the ATET/SEPQ quadruple mutant ookinetes. It is a simple experiment that if it worked would be informative. If it failed it might indicate that the defect is due to the inability of specific ABPs to bind to the quadruple mutant.

Authors' response: We thank the reviewer for this very helpful suggestion. We agree that this result would be informative and thus performed additional experiments for the revised version. We however conducted the experiments with hemolymph sporozoites instead of the suggested ookinetes for two reasons: (1) hemolymph sporozoites was the stage more studied in the paper and the findings from this experiment could have more direct relevance for other pre-salivary gland invaded mutants (as opposed to a potentially ookinete specific finding). (2) There is experimental precedent that such "chemical rescue" experiments have been successfully used with actin modulators with sporozoites (Hellmann et al 2013 Adv Healthcare Mater 2:1162). This gives us more confidence with the outcomes and interpretations of the experiments.

We used concentrations of Jasplakinolide previously reported to rescue the motility defect of a genetically modified sporozoite (Hellmann et al 2013 Adv Healthcare Mater 2:1162) and alter retrograde flow and force transduction of wild-type sporozoites (50 nM) (Quadt et al 2016 ACS Nano 10(2):2091, Munter et al 2009 Cell Host Microbe 6(6):551); and a higher concentration shown to affect sporozoite traction (100 nM) (Munter et al 2009 Cell Host Microbe 6(6):551). Neither of these concentrations rescued the motility defect of the ATET/SEPQ mutant, hinting that the modifications could be resulting in the inability of specific actin binding proteins to bind to the mutant.

We have included the results of this experiment as a supplementary figure (new Supplementary Figure 2) and added text in both the results and discussion sections (lines 208-212 and 539-543 in clean version) highlighting this finding and its potential implications.

Not a proposed experiment for this study, but in addition to changes in temperature optimization there could be different actin binding proteins functioning in mosquito stages. An interesting future experiment would be to use the chromobody in proximity labeling experiments. TurboID or another biotin ligase fused to the chromobody could be used to identify which proteins associate with actin in different Plasmodium stages. This same experiment in the ATET/SEPQ mutant would also directly test the idea that specific ABPs are no longer associating with actin filaments in the mosquito stages.

Authors' response: We thank the reviewer for this suggestion. We are indeed interested in characterizing differential actin binding protein actions and/or the role of parasite unique actin binding proteins. We will bear this approach in mind for future work.

Reviewer #2: Minor: 1. Figs. 2B, 2F, 3C, 3F make the individual data points smaller so they don't merge.

Authors' response: We have made these changes as suggested.

2. In Fig. 3B don't break the Y-axis (doesn't save that much space with the breaks you made and looks strange).

Authors' response: We tried an unbroken axis but it becomes quite difficult for the reader to see the changes in the motile population (one of the major points of the analysis). We

thus need a segmented axis. We have nonetheless altered the axis such that only one break point is necessary and this markedly improves the aesthetics of the panel.

Reviewer #3: 1. The actin localization in Figs. 5 and 6 are somewhat difficult to interpret. Without DIC/Nomarski images, the outlines of the parasites cannot be seen. For example, when actin localizes to both ends, is actin localized to the very ends or somewhere near the tips? Some of the representative images should be shown as overlays between fluorescent and DIC images to clearly demonstrate the locations of actin-rich regions.

Authors' response: We attempted to do this but high quality merges between channels are actually difficult to accomplish due to the very rapid motility of the sporozoites. It takes approximately 0.5-1 second to acquire two channels (due to the exposure times and technical acquisition rate limits of the microscope) and the sporozoites have moved on average about 2 microns in this time. This equates to approximately 15-20% of the total body length of the parasite, making it difficult to exactly distinguish between the absolute ends and proximity to the tips. Indeed, in previous work, to be more confident with co-localization of a protein of interest, we had to treat the sporozoites with Cytochalasin D to stop them moving, giving us more confidence of the protein location (Klug et al 2016 Open Biol 6(4):160034). Due to the sensitivity of the chromobody to Cytochalasin D, this is not possible for our current study (for untreated parasites). We have nonetheless added dotted lines to some panels as a best estimate to orient the reader. But we have been cautious to provide a detailed interpretation of the exact localization (for example, differentiating between "back" and "close to the back") due to the limitations highlighted above. Ultimately, we plan to do super-resolution (possibly coupled to expansion microscopy) imaging with some parasite lines that will be fixed prior to imaging to avoid the shift but this would go beyond the scope of the current study.

2. It would be helpful for readers if a table for figure summarizing the results of mutations and phenotypes.

Authors' response: We have included a summary panel as part of the revised figure 7. This addition nicely illustrates the pronounced effect of the different mutations across the life cycle, particularly on salivary gland invasion.

Appendix 2: Changes made to revised manuscript (Manuscript ID: PPATHOGENS-D-22-00139)

Please note: line numbers correspond to the clean manuscript version.

Textual changes

Lines 51-64: Addition of Author Summary.

Lines 101-103: Added text to introduction to include a recent BioRxiv paper.

Lines 160-165: Text added regarding residue interactions based on available structure.

Line 172: “Supp Figure 1” changed to “S1 Figure” to be consistent with PLOS formatting.

Lines 208-212: Text added regarding results obtained during revision (rescue experiment with Jasplakinolide and ATET/SEPQ mutant).

Line 265: “Supp Figure 3” changed to “S4 Figure” to be consistent with PLOS formatting. Changed to S4 to include new datasets in revised submission.

Lines 265-269: Text added regarding results obtained during revision (chromobody sporozoite analysis).

Line 284: “Supp Figure 4” changed to “S6 Figure” to be consistent with PLOS formatting. Changed to S6 to include new datasets in revision.

Lines 299-303: Text added regarding results obtained during revision (chromobody fluorescence analysis).

Lines 303-305: Minor textual edit to make sentence clearer. Sentence now reads: “While treatment of the mutant with Jas did not strikingly increase the number of sporozoites with “front and back” localization, treatment with CytoD abrogated the localization, just as it did in the wild-type.”

Lines 361-365: Addition of discussion text based on results obtained during revision.

Lines 391-395: Addition of discussion text based on reviewer comment.

Lines 405-409: Addition of discussion text based on results obtained during revision.

Lines 431-432: Minor textual edit to put sentence into future tense. The sentence now reads: “Further, the chromobody will be a valuable tool to study other stages of the *Plasmodium* life cycle.”

Lines 454-462: Addition of discussion text based on results obtained during revision.

Line 469: Numbers of pausing sporozoites mentioned in discussion updated to reflect fully counted dataset (as opposed to the initial count included for the initial submission). Overall result and interpretation remain the same and rest of the text remains unaltered.

Lines 539-543: Addition of discussion text based on results obtained during revision.

Lines 578-581: Additions of extra detail on ethics statement as requested by editorial team.

Lines 749-755: Addition of discussion text to describe methods used during revision experiments.

Lines 768-771: Addition of discussion text to describe methods used during revision experiments.

Lines 784-786: Addition of extra details for one previously mentioned funding body (SFB1129).

Figure and figure legend changes

Figure 1, panels A and B: Change of subunit colours and addition of boxes with insets. Figure legend updated to included these changes

Figure 2, panels B and F: circles representing data points made smaller to reduce overlap as suggested by the reviewer.

Figure 2, panel A legend: word “bracket” changed to “dashed line”.

Figure 2, panel B legend: word “infections” removed as pointed out by the reviewer.

Figure 3, panels C and F: circles representing data points made smaller to reduce overlap as suggested by the reviewer.

Figure 3, panel A legend: word “bracket” changed to “dashed line”.

Figure 3, panel G legend: “Supp Figure 2” changed to “S3 Figure” to be consistent with PLOS formatting. Changed to S3 to include new datasets in revised submission.

Figure 4, panel A legend: word “bracket” changed to “dashed line”.

Figure 5, panels A and B: Dotted lines added on left images of each panel to illustrate sporozoite outline as requested by reviewer 3.

Figure 5, panel B legend: Text added to indicate dotted lines for both panels A and B.

Figure 7: addition of new panel A to indicate phenotypes summary as suggested by reviewer 3. Figure legend amended to include this addition.

Figure 7, new panel B legend: Minor additions to the text to make explanation clearer.

S2 Figure and legend added to include dataset based on experiment suggested by the reviewer.

S5 Figure and legend added to include dataset based on experiment suggested by the reviewer.

S1 Data and S1 Table legends added.

References added to manuscript

Frenal K, Dubremetz JF, Lebrun M, Soldati-Favre D. Gliding motility powers invasion and egress in Apicomplexa. Nat Rev Microbiol. 2017;15(11):645-60. (now reference 20)

Hueschen et al (2022) Emergent Actin Flows Explain Diverse Parasite Gliding Modes. BioRxiv DOI: https://doi.org/10.1101/2022.06.08.495399 (now reference 21)

Hellmann et al (2014) Tunable substrates unveil chemical complementation of a genetic cell migration defect. Adv Healthcare Mater 2:1162 (now reference 42)
